# The circumventricular organs participate in the immunopathogenesis of experimental autoimmune encephalomyelitis

**DOI:** 10.1186/1743-8454-2-8

**Published:** 2005-09-30

**Authors:** Martina Schulz, Britta Engelhardt

**Affiliations:** 1 Theodor Kocher Institute, University of Bern, CH-3012 Bern, Switzerland; 2 Kerckhoff Institute, Department of Vascular Biology, Bad Nauheim, Germany; 3 Max-Planck Institute for Molecular Biomedicine, Münster, Germany

## Abstract

**Background:**

During inflammatory conditions of the central nervous system (CNS), such as in multiple sclerosis or in its animal model, experimental autoimmune encephalomyelitis (EAE), immune cells migrate from the blood stream into the CNS parenchyma and into the cerebrospinal fluid (CSF) spaces. The endothelial blood-brain barrier (BBB) has been considered the most obvious entry site for circulating immune cells. Recently, the choroid plexus has been considered as an alternative entry site for circulating lymphocytes into the CSF. The choroid plexus, belongs to the circumventricular organs (CVOs) localized in the walls of the ventricles. Other CVOs, which similar to the choroid plexus lack an endothelial BBB, have not been considered as possible entry sites for immune cells into the CNS parenchyma or the CSF. Here we asked, whether CVOs are involved in the recruitment of inflammatory cells into the brain during EAE.

**Methods:**

We performed an extensive immunohistological study on the area postrema (AP), the subfornical organ (SFO), the organum vasculosum of the lamina terminalis (OVLT) and the median eminence (ME) in frozen brain sections from healthy SJL mice and mice suffering from EAE. Expression of cell adhesion molecules, the presence of leukocyte subpopulations and the detection of major histocompatibility complex antigen expression was compared.

**Results:**

Similar changes were observed for all four CVOs included in this study. During EAE significantly increased numbers of CD45^+ ^leukocytes were detected within the four CVOs investigated, the majority of which stained positive for the macrophage markers F4/80 and Mac-1. The adhesion molecules ICAM-1 and VCAM-1 were upregulated on the fenestrated capillaries within the CVOs. A considerable upregulation of MHC class I throughout the CVOs and positive immunostaining for MHC class II on perivascular cells additionally documented the immune activation of the CVOs during EAE. A significant enrichment of inflammatory infiltrates was observed in close vicinity to the CVOs.

**Conclusion:**

Our data indicate that the CVOs are a site for the entry of immune cells into the CNS and CSF and consequently are involved in the inflammatory process in the CNS during EAE.

## Background

In multiple sclerosis and in its animal model, experimental autoimmune encephalomyelitis (EAE), inflammatory cells obtain access to the central nervous system (CNS) parenchyma and the cerebrospinal fluid (CSF) and initiate the events leading to signs of paralysis. The endothelial blood-brain barrier (BBB) has been considered the obvious place for entry for circulating lymphocytes into the CNS. Therefore most investigations have focused on defining the molecular mechanisms involved in leukocyte recruitment from the circulating blood across the endothelial BBB. The adhesion molecules, intercellular adhesion molecule 1 (ICAM-1) and vascular cell adhesion molecule 1 (VCAM-1), both members of the immunoglobulin superfamily, are upregulated on the endothelial cells of cerebral vessels during EAE and actively involved in the recruitment of inflammatory cells across the BBB (summarized in [1]).

Trafficking pathways for the entry of immune cells into the CSF remain unknown to date. The CSF of healthy individuals contains between 150,000 cells and 500,000 cells. During multiple sclerosis this number increases dramatically. Neither in the healthy individual nor during multiple sclerosis does the cellular composition of the CSF reflect that of the peripheral blood, suggesting a stringent control for leukocyte entry into the CSF at all times [2]. Recently it was considered that leukocytes enter the CSF using a direct pathway through the choroid plexus. The microvessels within the choroid plexus are different to those in brain parenchyma, the most significant of which is that the endothelial cells allow free movement of molecules via fenestrations and intercellular gaps (reviewed in [3]). Instead, the barrier is located at the level of the choroid plexus epithelial cells, which form tight junctions inhibiting paracellular diffusion of water soluble molecules [4].

Migration of leukocytes through the choroid plexus into the CSF has been suggested by the finding that fluorescently labeled splenocytes are present in the choroid plexus stroma two hours after intravenous injection in mice [5]. The adhesion molecules ICAM-1 and VCAM-1, which are required for leukocyte entry into the CNS, are expressed on the choroid plexus epithelium [6], become upregulated during EAE, and can mediate lymphocyte binding *in vitro *[7]. These observations suggest that the choroid plexus is involved in the communication of the immune system with the CNS probably by allowing the entry of immune cells directly into the CSF spaces.

Besides the choroid plexus there are additional structures in the CNS of mammals lacking an endothelial BBB. These areas fulfill neurohemal and neurosecretory functions, in that the neurons monitor hormonal stimuli and other substances within the blood or secrete neuroendocrines into the blood, and are commonly referred to as the circumventricular organs (CVOs; reviewed in [8,9]). CVOs are localized at strategic points close to the midline of the brain within the ependymal walls lining the 3^rd ^and the 4^th ^ventricle. Because they lack an endothelial BBB they lie within the blood milieu and thus form a blood-CSF barrier. At the cellular level the barrier between the CVOs and the neuropil is established by specialized epithelial cells called tanycytes. The median eminence (ME) belongs to the purely neuroendocrine CVOs also including the pineal gland and the neurohypophysis. The ME is localized between the stem of the infundibular or pituitary stalk of the hypophysis and the hypothalamus at the base of the 3^rd ^ventricle. The ME contains the terminations of axons from hypothalamic neurons and specialized glial cells. Neuroendocrine functions of the ME include the release of gonadotropin-releasing hormone by which the ME influences reproductive functions [10].

The sensory CVOs such as the subfornical organ (SFO), the organum vasculosum of the lamina terminalis (OVLT) and the area prostrema (AP) are characterized by a high density of small neurons, astrocytes and microglial cells [11]. The SFO and the OVLT are structures in the anterior wall of the 3^rd ^cerebral ventricle. The SFO bulges from the roof of the 3^rd ^ventricle into its lumen at the level of the interventricular foramina, whereas the OVLT is localized in the lamina terminalis between the chiasma opticum and the anterior commissure. The AP can be found at the caudal end of the *fossa rhomboidea *in the floor of the 4^th ^ventricle. The sensory CVOs monitor the changes in osmotic, ionic and hormonal composition of the blood and are therefore involved in regulating thirst and fluid metabolism [12].

The strategic localization of the CVOs in the wall of the ventricles prompted us to ask whether the CVOs might, similar to the choroid plexus, be involved in the recruitment of immune cells into the brain during EAE. We have performed an extensive immunohistological study localizing adhesion molecules, MHC class I and II antigens and leukocyte subpopulations within the subfornical organ (SFO), the median eminence (ME), the organum vasculosum of the lamina terminalis (OVLT) and the area postrema (AP) of the SJL/N mouse. During EAE similar changes were observed within all four CVOs included in this study. Specifically, the presence of significantly increased numbers of CD45^+ ^leukocytes in the CVOs suggested the recruitment of inflammatory cells into the the parenchyma of the CVOs probably mediated via endothelial ICAM-1 and VCAM-1 which were both upregulated on the microvessels within the CVOs. These changes were accompanied by a significant upregulation of MHC class I throughout the CVOs and induction of MHC class II on perivascular cells. Our observations indicate an active communication between the CVOs and the CNS parenchyma and thus the involvement of the CVOs in the inflammatory process of the CNS during EAE.

## Methods

### Induction of EAE

Female SJL/N mice were obtained from Bomholdgård Breeding, Ry, Denmark and used for experiments at the age of 10 weeks. Active EAE (aEAE) was induced by immunization with syngeneic spinal cord homogenate as described in detail before [13,14]. Briefly, SJL/N mice were immunized with 100 μg of spinal cord homogenate (SCH) from syngeneic mice in complete Freund's Adjuvant (CFA) (Gibco Laboratories, Grand Island, NY), containing 60 μg/ml *Mycobacterium tuberculosis *H37Ra and 10 μg/ml *Mycobacterium butyricum *(Difco Laboratories, Detroit, MI) subcutaneously. Heat-killed *Bordetella pertussis *organisms (3 × 10^9^; kindly provided by Dr. Kolbe, Behring Werke, Marburg, Germany or Dr. Kohler, Berna Biotech, Bern, Switzerland) were injected in 0.5 ml of PBS on days 1 and 3 post immunization. Animals were checked daily and clinical severity was documented as follows: 0.5 = limp tail; 1 = weak hindlimbs, unsteady gate; 2 = paraplegic, 3 = paraplegic plus incontinent and weakness. Clinical disease occurred approximately 14 days post immunization with spinal cord homogenate. A total of 46 mice with a clinical severity of EAE of 1 to 2 during their first clinical episode are presented in this study. As control, 10 untreated littermates, which were kept in the same cages – were sacrificed and investigated in the same way. All animal experiments were performed in accordance with the German and the Swiss legislation on the protection of animals and approved by the respective government authorities (permission numbers B2/127 and 55/04).

### Monoclonal Antibodies

Mec13.3 (anti-mouse PECAM-1/CD31), MK2.7 (anti-mouse VCAM-1), 3C4 (anti-mouse ICAM-2), RB40.34/4 (anti-mouse P-selectin), Lyt 2 (anti-mouse CD8), 145-2C11 (anti-mouse CD3), F4/80 (anti-mouse macrophages), FD441.8 (anti-mouse LFA-1), Mel-14 (anti mouse L-selectin) were purchased form BD Pharmingen, Germany, where detailed information on the antibodies can be obtained . C363 (anti mouse CD3ε) was purchased from Southern Biotechnology Associates, Birmingham, AL, USA, . ER-TR2 (rat- anti mouse MHC class II) and ER-MP42 (rat anti-mouse MHC class I) were purchased from Dianova, Hamburg, Germany, . The hybridomas PS/2 (anti-mouse α4-integrin), M1/9 (anti-mouse CD45), M1/70 (anti-mouse Mac-1), B220 (anti-mouse CD45R), GK1.5 (anti-mouse CD4), MECA-367 and MECA-89 (anti- mouse MAdCAM-1) and Hermes-1 (= 9B5, anti-human CD44, used as an isotype-matched control) were obtained from ATCC (Rockville, MA, USA; ). 25ZC7 (anti-mouse ICAM-1) and 9DB3 (anti-mouse VCAM-1) were kindly provided by D. Vestweber (Münster, Germany; [13]. UZ 4 and UZ 7 (both anti-mouse E-selectin) and MECA 32 were kindly provided by R. Hallmann (Münster, Germany; [14,15]).

### Immunohistochemistry

Animals were anesthetized using isoflurene anesthesia (Abbott, Wiesbaden, Germany), and were perfused with either phosphate buffered saline (PBS) or 1% formaldehyde (PFA) in PBS through the left ventricle of the heart. Tissue was removed, embedded in Tissue-tec, (OCT, Miles Inc., Vogel, Giessen, Germany), and snap-frozen in a 2-methylbutane (Merck, Darmstadt, Germany) bath at -80°C. Cryostat sections (6 μm) were air dried overnight, acetone fixed, and stained using a three-step immunoperoxidase technique. Sections were incubated sequentially with primary mAbs, biotinylated secondary goat-anti-rat IgG, (Vector, Boehringer Ingelheim Bioproducts, Heidelberg, Germany), and horseradish peroxidase-conjugated Streptavidin (Vector) for 30 min each step in a humidified chamber, with PBS washes in between the single steps. Sections were developed with 0.07% amino-ethylcarbazol (AEC, Sigma, Germany) and 0.009% hydrogen peroxide in 0.01 M acetate buffer (pH 5.2) for 10 min. Sections were counterstained with Hematoxylin (Gill's formula, Merck), coverslipped with Aquatex (Merck, Darmstadt, Germany) and immediately analyzed.

To detect presence of luminal antigens in cerebral blood vessels mice were injected intravenously with 250 μg primary antibody, anesthetized 15 or 30 minutes later, perfused first with PBS to remove unbound antibody then with 1% PFA to fix bound antibody. Tissue was processed as described above and immunohistology was performed accordingly leaving out the first antibody. This study summarizes results from a total of 49 individual immunostainings where CVOs from 46 EAE mice and 10 healthy mice were compared.

## Results

**Figure 1:**
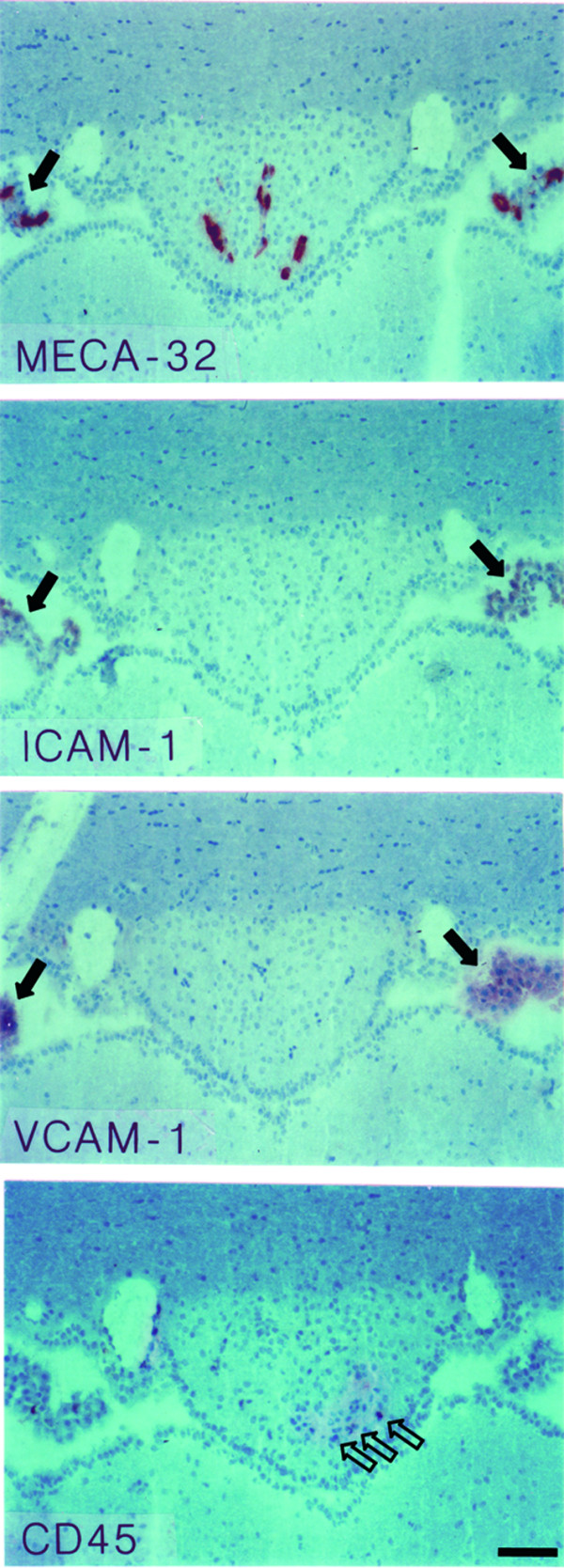
**Immunohistology of the subfornical organ (SFO) in the healthy SJL mouse**. The top panel shows MECA-32^+ ^capillaries within the SFO. Panels below demonstrate that ICAM-1 and VCAM-1 can not be detected on the MECA-32^+ ^capillaries within the SFO. Single CD45^+ ^perivascular cells can be detected within the SFO (open arrows, lower panel). Note the positive immunostaining for MECA-32 on choroid plexus capillaires and for ICAM-1 and VCAM-1 on the choroid plexus epithelium visible at the left and right margins (closed arrows). Immunoperoxidase, hematoxylin counterstain. Bar = 100 μm. This result was observed 10 times.

**Figure 2:**
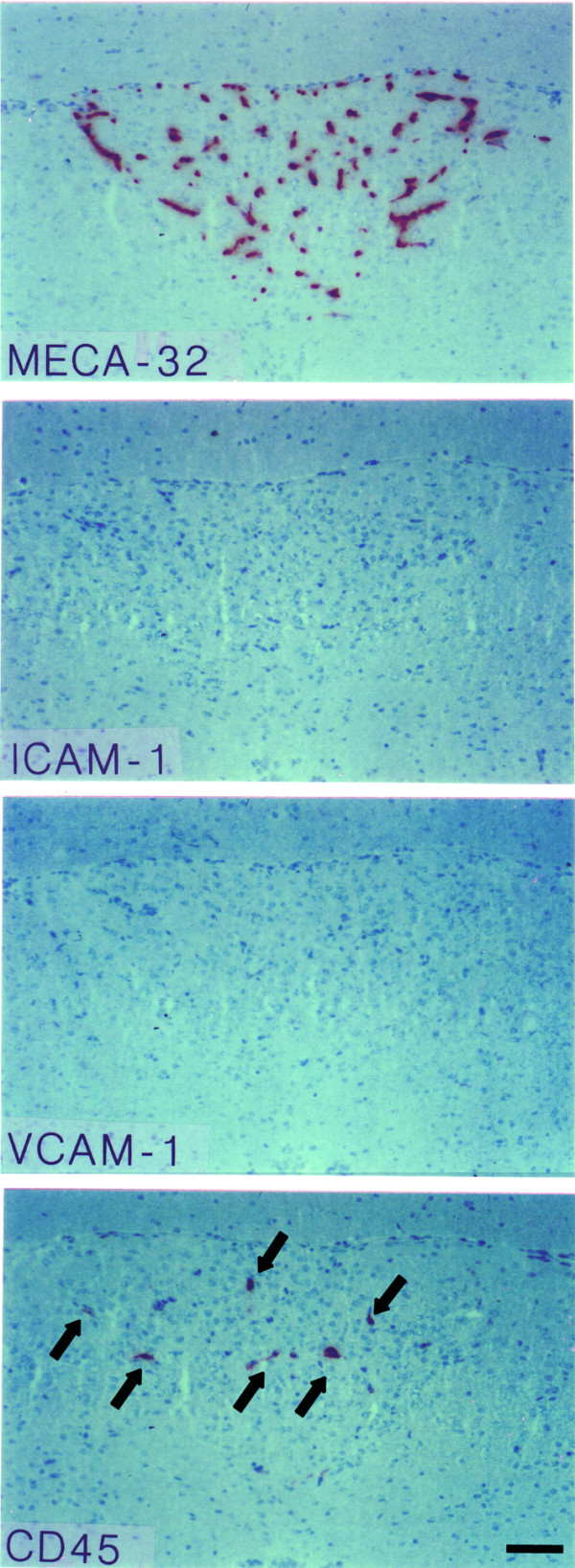
**Immunohistology of the area postrema (AP) in the healthy mouse**. The V-shaped area of the AP is outlined by the MECA-32^+ ^capillaries (top panel). ICAM-1 and VCAM-1 can not be detected on the MECA-32^+ ^capillaries within the AP. Single CD45^+ ^perivascular cells are present in this CVO (e.g. arrows, lower panel). Immunoperoxidase, hematoxylin counterstain. Bar = 100 μm. This result was observed 10 times.

**Figure 3:**
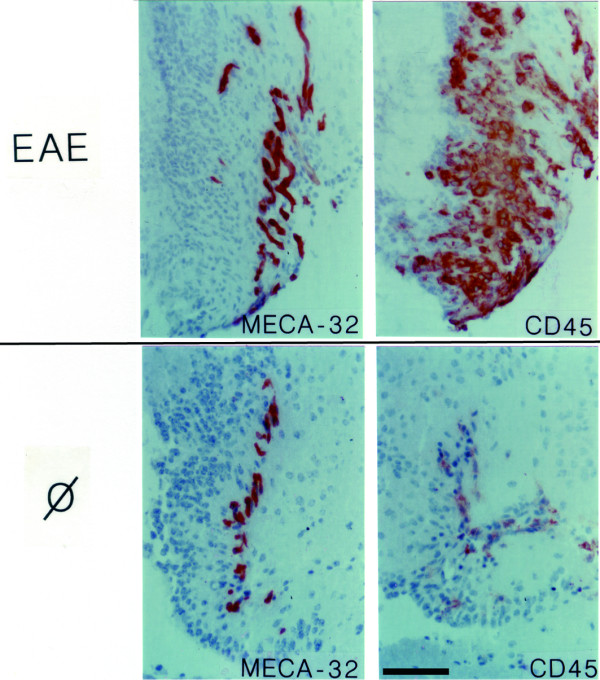
**CD45^+ ^cells in the organum vasculosum of the lamina terminalis (OVLT)**. Some CD45^+ ^cells, which are not exclusively associated with the MECA-32^+ ^capillaries can be detected within the OVLT of healthy SJL mice (lower two panels; Ø = healthy control). During EAE there is an increased number of CD45^+ ^cells present within the OVLT (upper two panels; EAE). Immunoperoxidase, hematoxylin counterstain. Bar = 100 μm. This result was observed in at least 10 individual stainings.

**Figure 4:**
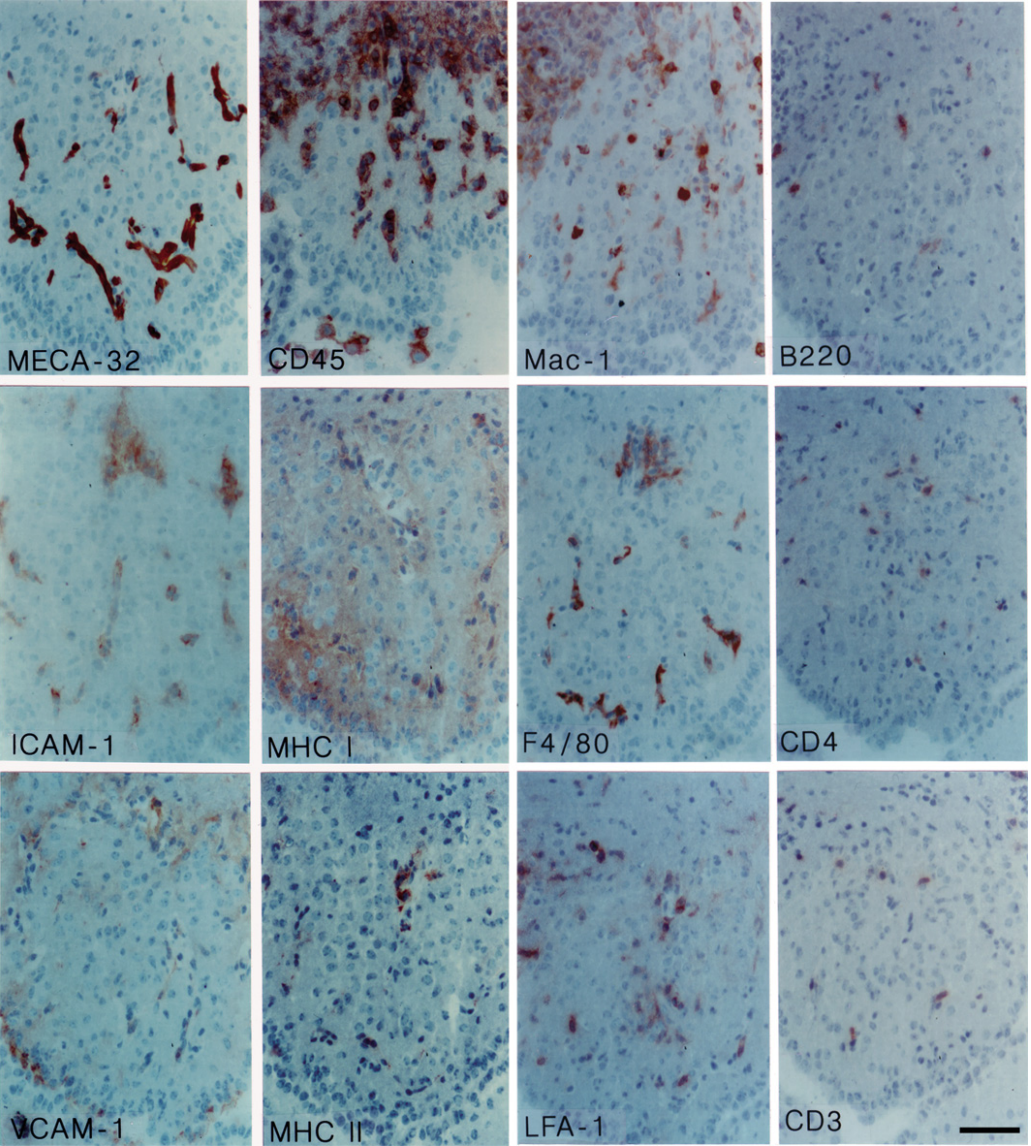
**Immunohistology of the subfornical organ (SFO) during EAE**. During EAE, MECA-32^+ ^capillaries within the SFO stain positive for ICAM-1 and VCAM-1 (left panels). A large number of CD45^+ ^cells preferentially localized in close vicinity to the microvessels can be detected within the SFO (second column top panel). Whereas expression of MHC class I is upregulated throughout the SFO parenchyma seen as diffuse staining, MHC class II is solely induced on perivascular cells (second column middle and lower panel). The majority of CD45^+ ^cells within the SFO during EAE are macrophages/microglial cells as determined by positive immunostaining for the β2-integrin Mac-1 and for F4/80 (third column top and middle panel). Note, whereas staining for Mac-1 can be detected on round and ramified cells throughout the SFO, staining for F4/80 remains restricted to perivascular cells. Besides macrophages, a low number of CD3^+ ^T lymphocytes of the CD4 and CD8 (data not shown) subclass and B220^+ ^B cells can be detected (right panels). Most of the immune cells present within the SFO stain positive for LFA-1, but negative for α4-integrins (not shown). Immunoperoxidase, hematoxylin counterstain. Bar = 100 μm. This result was observed in at least 10 individual stainings.

**Figure 5:**
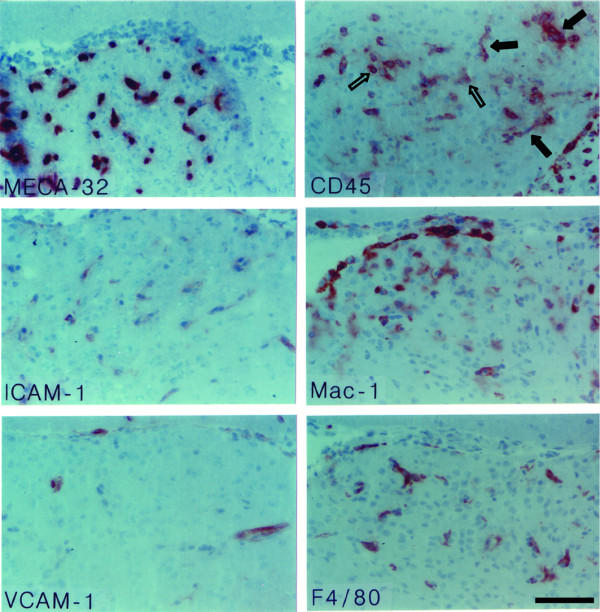
**Immunohistology of the area postrema (AP) during EAE**. MECA-32^+ ^capillaries within the AP stain positive for ICAM-1 and VCAM-1 during EAE. A large number of CD45^+ ^cells can be detected within the AP, which are associated with the microvessels (e.g. filled arrows) or localized within the parenchyma (e.g. open arrows). The majority of those are macrophages/microglial cells as determined by positive immunostaining for F4/80 and Mac-1. Note, whereas staining for Mac-1 can be detected on cells throughout the AP, staining of F4/80 is only seen on perivascular cells. Immunoperoxidase, hematoxylin counterstain. Bar = 100 μm. This result was observed in at least 10 individual stainings.

**Figure 6:**
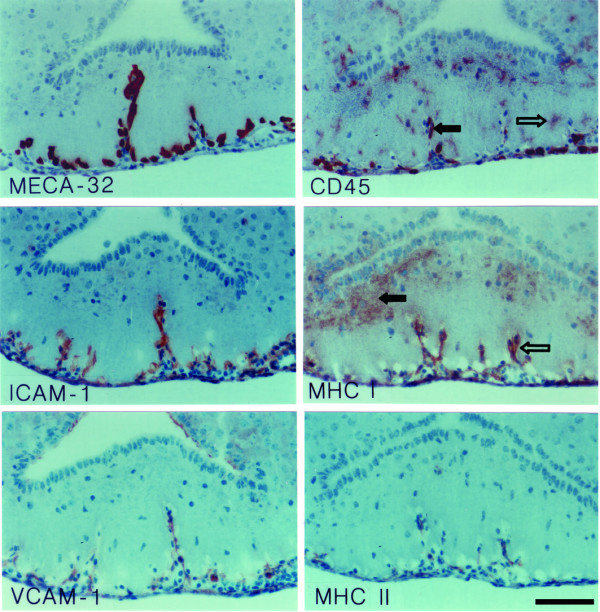
**Immunohistology of the median eminence (ME) during EAE**. MECA-32^+ ^capillaries within the ME stain positive for ICAM-1 and VCAM-1. Positive staining for CD45^+ ^can be detected throughout the ME, staining perivascular cells (examples marked by filled arrow) or parenchymal cells (example marked by open arrow). MHC class I is detectable within the entire ME, with parenchymal localization (closed arrows) as well as vessel associated (open arrow). MHC class II is only present on perivascular cells. Immunoperoxidase, hematoxylin counterstain. Bar = 100 μm. This result was observed in at least 10 individual stainings.

**Figure 7:**
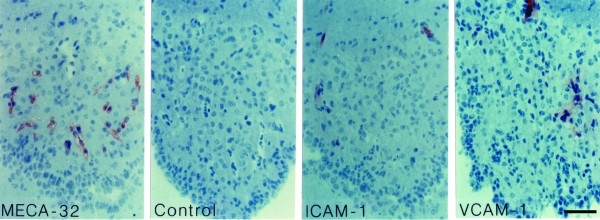
**Detection of luminal ICAM-1 and VCAM-1 in the capillaries of the SFO during EAE**. Mice suffering from EAE were perfused intravascularly with antibodies directed against MECA-32, ICAM-1 or VCAM-1; an irrelevant rat IgG was perfused as control. Bound antibody was detected by immunohistology. Only a subpopulation of capillaries within the SFO stain positive for luminal ICAM-1 and VCAM-1. Immunoperoxidase, hematoxylin counterstain. Bar = 100 μm. This result was observed 3 times.

**Figure 8:**
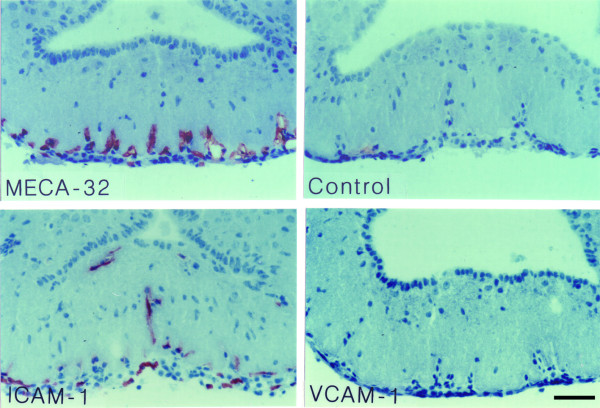
**Detection of luminal ICAM-1 and VCAM-1 in the capillaries of the ME during EAE**. Mice suffering from EAE were perfused intravascularly with antibodies directed against MECA-32, ICAM-1 or VCAM-1; an irrelevant rat IgG was perfused as control. Bound antibody was detected by immunohistology. Only ICAM-1, not VCAM-1, could be detected on the luminal surface of a subpopulation of capillaries in the ME during EAE. Immunoperoxidase, hematoxylin counterstain. Bar = 100 μm. This result was observed 3 times.

In the mouse the panendothelial antigen MECA-32 becomes specifically downregulated in brain endothelia during the maturation of the blood-brain barrier [15]. As a consequence, the MECA-32 antigen was absent on the mature cerebral endothelium, whereas it remained present on vessels outside of the CNS and the microvessels within the choroid plexus and the circumventricular organs (CVOs, Figures 1, 2, 3, 4, 5, 6, 7, 8).

In the present study we investigated four CVOs, namely the subfornical organ (SFO), the area postrema (AP), the median eminence (ME) and the organum vasculosum of the lamina terminalis (OVLT), which all lack an endothelial blood-brain barrier (BBB) and demonstrate fenestrated MECA-32^+ ^microvessels.

### The circumventricular organs in healthy SJL/N mice

In general, characteristics of the 4 CVOs analyzed were similar. As expected in the healthy SJL/N mice we detected MECA-32^+ ^microvessels within the SFO (Fig. 1), in the AP (Fig. 2) and the ME and OVLT (Table 1). Additionally, CVO microvessels stained positive for PECAM-1 and some of them stained for ICAM-2 (Table 1). Adhesion molecules such as ICAM-1 and VCAM-1 (Figs. 1, 2 and Table 1) or MAdCAM-1, E- and P-selectin (Table 1) could not be detected on the microvessels within the CVOs of healthy mice. A low number of CD45-positive perivascular cells was regularly observed in all CVOs (Figs. 1, 2, 3 and Table 1). In contrast, in only 1 or 2 out of 10 immunostainings performed for each CVO, were individual perivascular cells staining positive for macrophage markers such as F4/80 or Mac-1 or MHC class I or II and LFA-1 detected in CVOs of healthy mice (Table 1). This suggests a variable and rarely detectable presence of individual perivascular macrophages within the CVOs during health. In contrast, parenchymal staining for these molecules was never detected in the CVOs of healthy mice. Similarly, staining for CD3, CD4, CD8 or B220 was always negative in the CVOs of healthy mice (Table 1).

**Table 1:** Summary of the immunohistochemical characterization of CVOs in health and EAE

**Molecule**	**Healthy**^1^	**EAE**^2^
***Microvessels***		

MECA-32	+ on all vessels	+ on all vessels
PECAM-1	+ on all vessels	+ on all vessels
ICAM-2	+ on some vessels	+ on some vessels
ICAM-1	No staining	+ on most vessels luminally and abluminally/perivascular
VCAM-1	No staining	+ on few vessels luminally and abluminally or perivascular no luminal VCAM-1 detected in ME
MAdCAM-1	No staining	no staining
E-selectin	no staining	no staining
P-selectin	no staining	no staining

***Immune cells***		

CD45	+ on few perivascular cells	+ on many cells within the CVOs
CD3	no staining	+ on few round cells ca. 4 cells/section in SFO ca. 10 cells/section in AP
CD4	no staining	+ on few round cells
CD8	no staining	+ on few round cells
B220	no staining	+ on few round cells
F4/80	only rarely observed on single perivascular cells*	+ on many exclusively perivascular cells
Mac-1 (αM-integrin)	only rarely observed on single perivascular cells*	+ on round and branched cells located perivascularly and throughout the CVOs
LFA-1 (αL-integrin)	only rarely observed on single perivascular cells*	+ on round and branched cells located perivascularly and throughout the CVOs
α4-integrin	no staining	very faint staining on few round cells – not regularly detected
MHC class I	only rarely observed on few perivascular cells^§^	considerable staining throughout the CVOs; negative on endothelium
MHC class II	only rarely observed on single perivascular cells*	+ on many perivascular cells

^1^All four CVOs were analysed in a total of 10 healthy mice. Results therefore summarize 10 immunostainings. ^2^A total of 46 mice has been analyzed. Staining for each marker in each CVO was observed at least 10 times. If not stated differently, stainings were repeatedly observed in all CVOs analysed. *Single immunopositive cells were observed in only 1 or 2 of 10 immunostainings. ^§^Few immunopositive cells were observed in 2 out of 10 immunostainings.

### The circumventricular organs during EAE in SJL/N mice

During EAE the changes observed within the four CVOs analysed were overall very similar. The microvessels within the CVOs retained their characteristic staining for the MECA-32 antigen, PECAM-1 and ICAM-2 (Figs. 3, 4, 5, 6, 7, 8, Table 1). In contrast to healthy mice, a high number of CD45^+ ^cells could be detected within the CVOs (Fig. 3, 4, 5, 6), with the most prominent increase of CD45^+ ^cells detected within the OVLT (Fig. 3). CD45^+ ^cells within all four CVOs were localized in close proximity to microvessels and were also found within the CVO parenchyma. Besides the intense CD45 staining on cells of round appearance, a fainter immunostaining for CD45 was detected on cells with a more ramified appearance, indicating that, in addition to the recruitment of CD45^+ ^cells into these areas, CD45 might also be upregulated on the resident microglial cells within the CVOs (Fig. 4, 5, 6).

In order to further characterize the CD45^+ ^cells detected within the CVOs during EAE we performed immunostaining for T cell, B cell, and macrophage markers. A large number of F4/80^+ ^and Mac-1^+ ^macrophages or microglial cells were detected within the CVOs (Figs. 4, 5). Whereas F4/80^+ ^cells were exclusively detected adjacent to CVO microvessels, Mac-1 positive cells could also be found within the parenchyma of the different CVOs (Fig. 4 and 5). Mac-1 positive cells presented either as round cells or cells with many processes, suggesting that Mac-1 was detected on macrophages recruited into the CVOs and also upregulated on resident microglial cells. Besides macrophages, few B220^+ ^B lymphocytes as well as few CD3^+ ^T lymphocytes could regularly be detected within the CVOs during EAE, especially within the SFO (Fig. 4). Most of the CD3 positive T cells recruited into the SFO were CD4^+ ^T helper cells as indicated by the detection of mostly CD4^+ ^but rarely CD8^+ ^cells within this CVO during EAE (Fig. 4 and Table 1).

The adhesion molecules from the integrin family, namely leukocyte function-associated antigen (LFA)-1 (αLβ2-integrin) and α4-integrins can be involved in inflammatory cell recruitment across the BBB during EAE. Therefore we investigated, whether CD45^+ ^cells localized within the CVOs also express these adhesion molecules. Whereas a high number of LFA-1^+ ^cells was detected in the CVOs (Fig. 4), α4-integrin positive cells were detected only rarely (Table 1).

Next we asked whether the potential endothelial ligands for Mac-1, LFA-1 and α4-integrins, namely intercellular adhesion molecule (ICAM)-1 and vascular cell adhesion molecule (VCAM)-1, are expressed on the microvascular endothelial cells within the CVOs. We found prominent induction of ICAM-1 and to a lesser degree of VCAM-1 (Figs. 4, 5, 6), but not of mucosal addressin cell adhesion molecule (MAdCAM)-1, E-selectin or P-selectin (Table 1) on CVO microvessels during EAE. In order to evaluate, whether ICAM-1 and VCAM-1 are available on the luminal surface of the CVOs capillary endothelial cells for the recruitment of circulating Mac-1^+^, LFA-1^+ ^and α4-integrin^+ ^leukocytes into the CVOs, we injected anti-ICAM-1 or anti-VCAM-1 antibodies into live animals suffering from EAE and investigated their binding to CVO microvessels by immunohistology. Whereas ICAM-1 was detected on the luminal surface of the capillaries of all CVOs investigated (Figs. 7, 8 and data not shown), VCAM-1 although present on the luminal surface of microvessels in the SFO, OVLT and AP (Fig. 7 and data not shown) could not be detected on the luminal surface of the capillaries within the ME (Fig. 8). Taken together, these observations suggest that recruitment of inflammatory cells into the CVOs mainly depend on LFA-1/ICAM-1 or Mac-1/ICAM-1 rather than VCAM-1/α4-integrin-mediated interactions.

In addition to the increased number of immunocompetent cells present in the CVOs during EAE, we observed a significant upregulation of major histocompatibility complex (MHC) antigens class I on cells throughout the entire parenchyma of the CVOs, where expression was absent before in healthy mice (Figs. 4, 6 and Table 1). In contrast, expression of MHC class II was solely induced on perivascular cells, a significant number of which could now regularly be found within all four CVOs during EAE (Fig. 4, 6 and Table 1).

**Figure 9:**
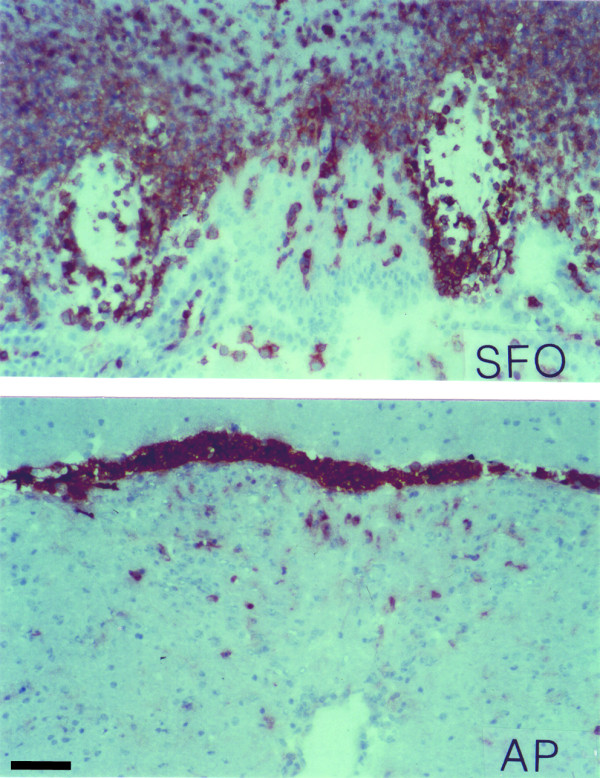
**Localization of inflammatory cell infiltrates in the brain of mice suffering from EAE are enriched in close vicinity to CVOs**. During EAE, CD45^+ ^cellular infiltrates in the brain were found to be enriched in close vicinity to CVOs. The top panel shows the SFO with perivascular CD45^+ ^cells present within the CVO. Massive CD45^+ ^infiltrates are prominent within the ventricular walls and the brain parenchyma outside but in close vicinity to SFO. The bottom panel shows the AP with perivascular and parenchymal CD45^+ ^cells present within this CVO. Many CD45^+ ^cells have accumulated at the border between the AP and the 4^th ^ventricle. These pictures are representative for the finding that CD45^+ ^inflammatory cell infiltrates in the brain were found to be enriched in number and size in close vicinity to CVOs during EAE. Immunoperoxidase, hematoxylin counterstain. Bar = 100 μm. This result was observed in at least 10 individual stainings.

Finally, besides the leukocyte infiltration of the CVOs proper, we observed the preferential localization of massive inflammatory cuffs within the brain parenchyma or the ventricular walls localized in close vicinity or directly adjacent to CVOs, suggesting an involvement of the CVOs in immune cell recruitment into these distinct areas (Fig. 9).

## Discussion

In the present study we provide evidence that immune cell invasion into the CNS during EAE is accompanied by a prominent immune activation of the CVOs. Changes within the CVOs are characterized by the presence of an increased number of CD45^+ ^cells, the considerable induction of MHC class I and MHC class II molecules within the CVOs as well as microglial activation documented by increased expression of Mac-1. Upregulated expression of adhesion molecules on the CVO microvessels suggest their involvement in the recruitment of macrophages, T- and B cells into the CVOs during EAE. Taken together, these observations indicate an active participation of the CVOs in the immunopathogenesis of EAE.

The involvement of CVOs in the communication of the immune system with the nervous system has been considered before. Due to their fenestrated capillaires the CVOs are often referred to as "windows of the brain" and have been thought to serve as entry points for pro-inflammatory cytokines into the CNS [16]. In fact, receptors for inflammatory cytokines and bacterial fragments are constitutively expressed in cells within the sensory CVOs and the release of pro-inflammatory cytokines such as interleukin-1 or TNF-α outside the CNS has been demonstrated to deliver signals into the CNS via the CVOs leading to neuroendocrine responses such as elevations in adrenocorticotropic hormone (ACTH) in the plasma, development of fever and the activation of the hypothalamic-pituitary-adrenal (HPA) axis [17]. Additionally, CVOs play an active role in the development of brain dysfunction during sepsis. Induction of experimental endotoxin shock by injection of lipopolysaccaride induces the sequential expression of molecules involved in innate immune responses such as CD14 or Toll-like receptors first in the CVOs and subsequently in the brain parencyhma (reviewed by [18]). Furthermore, involvement of CVOs in the establishment of an acute phase response during peripheral immune stimuli was demonstrated [19]. Finally, a role for CVOs in sending signals into the brain during systemic inflammation is supported by observations that lesions of CVOs block several components of brain-controlled illness responses (i.e. fever or neuroendocrine modifications) [20].

An active participation of CVOs in the auto-immunopathogenesis in EAE has not been reported to date. In the present study, we found that CVOs in the healthy SJL/N mouse presented a low number of perivascular CD45^+ ^cells, probably macrophages, similar to other regions of the CNS. We only occasionally detected expression of MHC class I or MHC class II on individual perivascular cells suggesting that MHC-positive cells rarely reside within CVOs of healthy mice. Adhesion molecules associated with inflammation could not be detected. In contrast, during clinical EAE at the time when immune cells have penetrated the BBB and perivascular infiltration of T cells and macrophages is observed within the CNS parenchyma, the CVOs demonstrated a dramatic increase in the presence of CD45^+ ^cells. As CD45 is upregulated on activated microglial cells, which reside in high numbers in CVOs [21], the increased number of CD45^+ ^cells detected within the CVOs during EAE is most probably due to both the recruitment of circulating CD45^+ ^immune cells from the periphery into these areas and additionally to the upregulation of CD45 on microglial cells residing within the CVOs.

Most of the CD45^+ ^cells also expressed the macrophage marker F4/80 or Mac-1, which can be expressed on macrophages or activated microglial cells. Based on the immunological and morphological criteria, these cells most probably can be classified as perivascular macrophages or ramified microglial cells, as classical parenchymal microglial cells, as well as scattered rounded macrophage-like cells. Activation of microglial cells by upregulated expression of CD45 and induction of Mac-1 therefore parallels the events observed within the CNS parenchyma protected by the BBB during EAE.

Microglial activation within the CVOs during EAE is further supported by our observation that MHC class I antigens were induced throughout the BBB-deficient parenchyma in all CVOs. In addition, MHC class II molecules could now regularly be detected on perivascular microglial cells or macrophages within the CVOs, which may function as a cellular barrier against blood-borne pathogens. Thus, increased expression of MHC class I and II molecules on microglial cells and perivascular macrophages, which are known characteristic immunopathological changes observed in the CNS parenchyma during EAE, are similarly observed in the BBB-deficient areas of the CVOs [22].

Besides the immune activation of the CVOs, recruitment of a low number of CD3^+ ^T cells and B220^+ ^B cells was detected in the parencyhma of CVOs during EAE, demonstrating that immune cells are invading these areas during EAE. It has been shown that endothelial VCAM-1 and ICAM-1 are involved in lymphocyte recruitment across brain endothelium *in vitro *and *in vivo *[13,23-25]. The upregulated expression of ICAM-1 and VCAM-1 on the microvessels of the CVOs during EAE suggests that the same molecules mediating immune cell entry across the BBB might be involved in guiding inflammatory cells into the CVOs. This notion was confirmed by our findings that both molecules are available on the luminal surface of CVO microvessels, with the exception of the median eminence, where luminal VCAM-1 could not be detected. Inflammatory cells within the CVOs were found to stain positive for Mac-1^+ ^and LFA-1^+^, therefore resemling those, present in the inflammatory cuffs surrounding brain venules forming a barrier [26]. In contrast, whereas during EAE inflammatory cells within the brain express high levels of α4-integrins [26] only few α4-integrin^+ ^leukocytes were found within the CVOs. These observations indicate that inflammatory cell recruitment into the CVOs is not necessarily mediated by the same mechanisms as those regulating leukocyte recruitment across the inflamed BBB, which during EAE was shown to be mainly dependent on α4-integrin and VCAM-1[1,25].

Whether immune cells enter the brain or CSF via the CVOs cannot be determined by this study. It is, however, tempting to speculate that CVOs have an active role in determining inflammatory cell recruitment into the brain as we observed a significantly increased number of inflammatory cuffs and infiltrating cells in close vicinity to the CVOs as compared to regions of the brain with a greater distance to the CVOs. These observations suggest that during EAE the inflamed CVOs deliver cellular or molecular signals into the CNS, which may influence immune cell entry into the CNS and CSF.

## Conclusion

Taken together our observations demonstrate that the CVOs are involved in the immunopathogenesis of EAE. Considering their strategic localization within the walls of the ventricles, they may be involved in immune cell entry from the blood into the CNS parenchmyma and also from the blood into the CSF. Therefore, the development of neuroprotective strategies will require consideration of the molecular changes in the CVOs during CNS inflammation.

## List of abbreviations

AP area postrema

BBB blood brain barrier

CD cluster of differentiation

CNS central nervous system

CVOs circumventricular organs

EAE experimental autoimmune encephalomyelitis

ICAM-1 intercellular adhesion molecule-1

LFA-1 leukocyte function associated antigen-1

MAdCAM-1 mucosal addressin cell adhesion molecule-1

ME median eminence

MHC major histocompatibility complex

OVLT organum vasculosum of the lamina terminalis

PECAM-1 platelet endothelial cell adhesion molecule -1

SFO subfornical organ

VCAM-1 vascular cell adhesion molecule -1

## Competing interests

The author(s) declare that they have no competing interests.

## Authors' contributions

The entire experimental work described in this paper was performed by MS. Evaluation of the data as well as the documentation was performed by both authors. BE designed and supervised the study, and wrote the manuscript. Both authors have read and approved the final manuscript.
